# A Collection of Cases of Apoplexy, with an Explanatory Introduction

**Published:** 1845-07

**Authors:** 


### A Collection of Cases of Apoplexy, with an Explanatory Introduction. By *Edward Copeman*, Surgeon. 8vo. pp. 205. London: Churchill, 1845.

The scope and object of this work are thus distinctly explained by the author. "The following collection of cases is published with the view of furnishing sufficient data for determining the comparative merits of different modes of treating Apoplexy, and for judging of the expediency of resorting to bleeding for the cure of that disease. It has long been my opinion that the popular, as well as professional, prejudice in favour of bleeding in affections of the brain is not justified by the result of the practice: and in order to convince myself, I collected the following cases. They are transcribed from various books and journals; a few are from my own case book; and I have purposely avoided introducing any author's remarks or comments, that each person who examines them may form an unbiassed opinion."

Before we proceed to give some account of its contents, it may be as well to recall to our readers' minds that the term Apoplexy simply indicates a particular assemblage of symptoms—the most prominent of which is a sudden loss of consciousness and voluntary motion, the breathing and circulation continuing—but does not suggest any idea as to the cause of these symptoms, or even as to the condition of the system in which they may occur; whether this be one of fulness or of debility, of plethora or of exhaustion. And yet it is but too true that many medical men are apt, on all occasions, to associate the idea of an apoplectic seizure with an engorged state of the cerebral bloodvessels, and thus are led to regard the detraction of blood as almost invariably necessary for the removal of the oppression, thereby induced. This, once common, mistake has indeed been corrected in all the standard treatises on the Practice of Medicine that have appeared within the last twenty years; but still there is a lurking tendency with many practitioners to resort, too indiscriminately, to bloodletting, whenever a person falls down in what is called a *fit*. With a view of exposing the fallacy and danger of this practice, Mr. Copeman has collected together no fewer than 250 cases, extracted from various works; and, although we must regret that they have not been arranged upon some definite plan, for the sake of convenient reference, the catalogue cannot fail to prove useful to the bibliographical student.

The author has scarcely done himself justice, we think, in not having extended his "introductory remarks;" for the impression, left upon our mind by their perusal, is that he is well acquainted with his subject, and fully competent to have treated it at greater length.

After pointing out that all *ages* are liable to attacks of Apoplexy, but that males are much more frequently affected than females (out of his 250 cases, 170 occurred in males), he makes the following useful remarks on the pathology of the disease:

"The morbid appearances found in persons who have died of apoplexy are exceedingly diversified, and by no means favour the idea that fulness of blood or rupture of a vessel is always the cause of death. The number of deaths in which the *post mortem* appearances are recorded is 156, and in 81 only was there extravasation of blood in the brain. In 10 cases there was found no diseased appearance of the brain at all, and in the remaining 65, the morbid appearances were, hypertrophy of the heart, disease of the aorta, softening of the brain, effusion of serum into the ventricles, or between the membranes, congestion of vessels in the brain, hydatids in the brain, inflammation of the brain, ossified arteries, abscess of the brain, diseased kidneys, brain hardened, cerebral vessels varicose, tubercle in the cerebellum, inflamed stomach, indurated liver, inflammation and ulceration of the small intestines, tumour in the brain." 4.

As to the prognosis in Apoplexy, we need scarcely say that very generally it must be decidedly unfavourable.

Of the 250 cases, 68 only recovered; 7 partially recovered; and 175 died—a very great mortality certainly; proving, as our author observes, "either that the proximate causes of the disease are beyond the reach of art, or that the measures usually adopted as remedies are inapplicable, inefficient, or prejudicial."

The following statement, and the practical deductions which are drawn from it, well deserve attentive consideration; they suggest a very useful practical lesson.

"The universal *remedy*, as it is called, for apoplexy is bloodletting; at least so generally has it been employed, that of 155 cases in which the treatment is specified, 129 were bled, and only 26 were not: of the 129 who were bled, 51 recovered and 78 died—the cures being 1 in 2½, the deaths 1 in 1⅗,—of the 26 who were not bled, 18 were cured and 8 died, the proportion of cures being 1 in 1½, and of deaths 1 in 3¼. But the mortality varies a good deal according to the particular method in which bloodletting was performed. In 2 cases the temporal artery was opened, both died. In 11 cases, cupping only was employd; 6 were cured and 5 died. Fourteen were treated by leeching; 4 cured, 10 died. Seventeen were bled in the foot, a plan strongly recommended by M. Portal; of which 13 were cured, and 4 died. Eighty-five were bled generally and copiously, of which number 28 recovered and 57 died; that is to say, two in every three cases terminated fatally. If we subtract the number of cases treated by bleeding in the foot, a plan of bleeding mild in its operation, and acting, probably, more on the principle of derivation than of materially lessening the quantity of blood in the system, we shall find that of 112 cases, 38 recovered and 74 died; reducing the proportion of cures to about 1 in 3, and increasing the proportion of deaths to 1 in 1½.

"From these facts it appears, that bleeding, generally speaking, is so ineffectual a means of preventing the fatal termination of apoplexy, that it scarcely deserves the name of a *remedy* for this disease. That where the temporal artery is opened the case terminates fatally; but as there are only two cases reported in which this method was exclusively employed, there are not sufficient data for establishing the universal fatality of this mode of treatment: although it may be fair to conjecture, that it is by no means desirable to abstract arterial blood from the brain. That bleeding in the foot was the most successful mode of abstracting blood. That the treatment without loss of blood was attended with the most success; and that the mortality of the disease increased in proportion to the extent to which the bleeding was carried; the more copious the loss of blood, the more fatal the disease." 7.

Even in cases, where we have reason to suspect extravasation of blood within the cranium, the practice of having immediate recourse to bloodletting is of very questionable propriety in many instances: "the mischief is done, the blood is effused, the system has received a great shock, and bleeding will not remove the extravasated blood." This is perfectly just; and certainly, unless we have reason to believe that there is co-existent arterial over-action or great venous engorgement, the mere suspected presence of a sanguineous coagulum on the surface, or within the substance, of the brain, is not *per se* a sufficient ground for a copious abstraction of blood. Mr. Copeman suggests that the extravasation may be promoted, rather than counteracted, by the greater thinness of the blood, and its diminished tendency to coagulate, induced by large bleedings. However this may be, it appears to be both safer and more judicious practice to allow (in some instances, at least) the system to recover from the immediate shock of the attack, before we lower the system by depletion. Much judgment, and not a little firmness and resolution too, will often be required, on the part of the medical man, to resist the wishes and expectations of the attendants, and to refuse to follow a plan that is almost universally followed, that of immediately opening a vein in the arm, and drawing blood, no matter what the concomitant symptoms may be, whether they indicate over-action or exhaustion. In many of the cases adduced by our author, there was a manifest deterioration of the symptoms—of the paralysis more particularly—after the patients had been copiously bled.

Mr. Copeman suggests the following sensible directions to guide the practitioner as to the plan he should adopt in the treatment of an apoplectic seizure.

"Under some states of constitutional disturbance, there may be, and undoubtedly is, a disposition for blood to be sent to the head, and an effort made to overfill the vessels of the brain; but when this is the case, I believe it will always be apparent by visible distension of the external vessels of the head and neck; there is so much obstruction to the entrance of more blood within the skull, and such free communication between the internal and external blood-vessels of the head, that when there is a disposition to greater fulness within, the external vessels will act as safety-valves, and there will be evident distension without. I therefore consider it as a practical rule, that if the vessels of the brain are unduly distended, it will be evidenced by fulness of the external vessels of the head; and that, unless we discover the latter, we are not justified in drawing blood for the relief of the supposed existence of the former. In my own opinion, the only cases of apoplexy in which bleeding is proper, are those which occur in plethoric habits, and where, in addition to the symptoms of what is generally understood by a full habit, there is evident distension or fulness of the superficial vessels of the head and neck. Neither do I consider that in these cases, unless there be inflammation of the brain, (which I apprehend is rarely the case,) it is necessary to carry the bleeding beyond the point of relieving entirely the external visible fulness of vessels, or congestion of the lungs from impeded circulation; beyond this we weaken the body by abstraction of blood, without lessening the quantity circulating in the brain; the vessels of the brain must always be full, and when we have removed the tendency to force an undue quantity of blood into them, we have done all we are required to do as a relief from pressure." 12.

He considers that the state of the pulse is a very uncertain criterion of the necessity of bleeding in Apoplexy. This opinion is, to a certain extent, quite true; and yet we must never omit to ascertain the force of the circulation, before determining what course of treatment to pursue. It may be worthy of notice here, that the strength of the heart's impulse is a much safer guide in this respect than the condition of the arterial pulse: and indeed it may be laid down as a general rule, that in all cases of disease, where there is any room for doubt as to the propriety of bloodletting, the practitioner should invariably examine the state of the heart, with his ear as well as with his hand. Were this simple plan universally followed, much serious mischief might be avoided in the treatment not only of Apoplexy but of many other disorders.

"When serious extravasation of blood has taken place, or when apoplexy occurs as the result of organic diseases of the brain, such as tumour, softening, abscess, &c., external signs of fulness are seldom present; indeed we may state that, in a great majority of such cases, the symptoms are strongly indicative of a prostrated condition of the system—the pulse is slow, sometimes intermitting; it may be either small or full, but its fulness is more like what we might suppose to arise, from the heart making efforts to propel the blood through passive tubes which had lost their natural elasticity for want of nervous influence; it is what I should rather call a *large* pulse; the countenance is often pale and covered with cold perspiration; the extremities are cold, the muscular system relaxed; the breathing laboured, and nervous power generally almost destroyed. I have myself observed, but perhaps it requires further confirmation, that the pupils being at first very much contracted and afterwards as much dilated, is a sign of fatal apoplexy." 13.

The leading therapeutic inference which Mr. Copeman draws from the 250 cases, whose histories he has collected together, is, that the chances of recovery from an apoplectic seizure are decidedly greater when general bloodletting is not employed. In not a few of them, where the attack supervened upon a full meal, the use of emetics was fouud to be strikingly beneficial. In some, where the powers of the system were visibly much depressed, the administration of stimulants, internal as well as external, powerfully contributed to remove the alarming symptoms; a blister or sinapism to the nape of the neck, and repeated doses of Ammonia, if the patient retains the power to swallow, are often of singular benefit. Purgatives appear to have been of essential service in most of the cases that recovered. Of this class of remedies, Croton oil and turpentine enemata are perhaps, on the whole, the best adapted to apoplectic cases.

We shall now select two or three of what appear to us the most interesting examples of the truth of these remarks.

CASE.—*Apoplectic attack; alarming state; sudden recovery after vomiting a large quantity of fœtid bile.*

"A gentleman, who had long shown symptoms of what would have been termed 'Ramollissement de Cerveau,' fell down in a fit of apoplexy, at the age of 68, and not the slightest impression was made by cupping, leeching, blisters, enemas, and all the means which a trio of physicians could suggest. One left the patient for dead, after taking four ounces of blood from the head; and he was apparently in articulo mortis, after 48 hours of general paralysis, total insensibility, stertorous breathing, glassy eyes, and dead 'rattles' in the throat! The physician took his leave at twelve o'clock at night, requesting to be informed in the morning at what hour the patient died. No message having been sent, the physician called in the morning, and found, to his no small surprise, the patient at his breakfast, quite sensible and with the full power of all his muscles! The patient soon after this, disgorged some pints of fetid bile, and had no return of apoplectic or paralytic symptoms.—*Medico-Chir. Review.* Vol. viii. p. 455."

In the following very interesting report—which well displays the quickness of perception and great practical sagacity of Dr. Johnson—the attack seems to have been conneccted with a retrocedent or misplaced gouty affection.

CASE.—*Insensibility and immobility followed by violent tremors; great tenderness of the epigastrium; partial consciousness after bleeding; great relief after vomiting.*

"I was requested to visit a gentleman said to be in a dying state. I found him apparently without sensation, his eyes opened and turned up, his hands clenched, and his body alternately motionless, and agitated by sudden and universal tremors, which caused the bed to shake beneath him. He was a stranger, and there was no one present who could give a history of his case. In order to explore into its nature, I placed my hand upon his epigastrium; but scarcely had I touched the skin when he started up as though a bullet had been driven through him. Uncertain whether the coincidence might not be accidental, I repeated the experiment several times, and at each time the slightest pressure was sufficient to throw the whole frame into immediate and violent, though brief convulsions. Sufficient evidence was thus afforded of the seat of the disease, but not of the precise nature of the irritation. As his pulse was active, I bled him freely, and immediately afterwards applied a large sinapism over the region of his stomach. Consciousness was so far restored a few minutes after the bleeding, that upon being asked in a loud voice if he felt sickness or pain in the stomach, he nodded in the affirmative. An injection of assafœtida was now administered; and under the united influence of this remedy and the mustard plaister, he revived to some knowledge of his situation, and was able to drink very freely of warm water, which I urged upon him. This soon produced the discharge of a large quantity of acid liquors from his stomach, and restored him for a time to complete consciousness. He now told me that he was subject to gout, of which he had recently an attack in his foot, but had relieved himself by bathing the affected part with hot vinegar. The nature of the case was evident. While he was yet speaking, he was seized with a sudden spasm of the stomach, which threw him into his former state; and this alternation of consciousness and insensibility was repeated several times within the course of a few minutes, each return of pain being so severe as at first to throw the whole nervous system into violent agitation, and then to overwhelm it for a time in complete torpor. I now applied sinapisms to the feet, and gave a mixture of laudanum and the aromatic spirits of ammonia; and at the end of about four hours from the commencement of the attack, left my patient very greatly relieved. A dose of the compound tincture of rhubarb, with a proper regulation of the diet, was afterwards sufficient to complete the cure.—*Medico-Chir. Review,* vol. xii. pp. 251 and 252." 44.

The next example we select is from Dr. Abercrombie's classical work on Diseases of the Brain: venesection was practised, the pulse being "of good strength and a little frequent."

CASE.—*Second Apoplectic attack after an interval of four years in a patient upwards of* 80 *years of age; coma alternating with covulsions; bleeding from the arm, cold to the head, and purgatives; recovery.*

"A lady, aged 82, on the morning of Sunday, 8th of March, 1818, complained of headache, but went to church. While in church she lost her recollection, talked incoherently, and was brought home with difficulty, being unable to stand. She was still incoherent, and partially comatose; and when put to bed was seized with violent convulsion, which affected chiefly her face and the left side of her body. The convulsions recurred frequently, leaving her in the intervals in a state of profound coma, and the left side appeared to be paralytic. The pulse was of good strength, and a little frequent. She was bled to twenty ounces; cold was applied to her head, and an active purgative was given as soon as she could swallow. On the following day there was little change; more purgative medicine was given. On the 10th the coma was diminished, but it was succeeded by much unmanageable restlessness with incoherence and some convulsion; pulse 112. More purgative medicine was given; and small doses of the tartrate of antimony seemed to be very beneficial. On the 11th there was little change, but on the 12th she was much improved, began to know her friends, and her pulse was coming down. In a few days more she was in her usual health, and lived for several years. This lady had also suffered an apoplectic attack in 1814." 80.

The actual pathological state or cause of death in the following case, it is not easy to determine by the symptoms during life; and, as there was no examination after death, there will probably be some difference of opinion respecting it.

CASE.—*Doubful seizure; the symptoms partaking more of the character of syncope from heart-disease than of apoplexy; death.*

"On the 25th December, 1806, a corpulent and robust man, the master of a Berwick smack, in going aboard his vessel, fell into the harbour. He was under water nearly a minute, and with some difficulty he got on board: but he was apparently so little injured, that he first changed his clothes in his cabin, and then walked home. I was sent for by him at one o'clock, shortly after the accident happened, and found him chilly, but not complaining, unless slightly of *his left* shoulder; his pulse was not affected; he *was rather faint;* he appeared so little injured, that nothing seemed necessary but to order him to bed, the sooner to restore the heat, and to give him some warm wine and water. I was again sent for at three o'clock. He was lying upon his right side, his head, from choice, low, and he was breathing rather hurriedly, and had a constant short cough: his extremities were cold; his pulse, at the wrist, was distinct; his countenance was of a leaden paleness; his lips were livid. He complained of considerable uneasiness in his chest, immediately under the left nipple, and of the pain of his shoulder. An attempt had been made, before I arrived, to draw blood both from the right and left arm: but he told us, that surgeons on various occasions had in vain attempted to draw blood from him. I perceived that he was not without a degree of stupor; and, as his breathing was becoming more hurried, I had the temporal artery opened, and the blood jetted from it darker than I ever saw venous blood. When about two cupfuls were procured, he complained of the inconvenience of the blood trickling into his eyes. I assisted him in turning to his left side, and his breathing became irregular, and stertorous; colourless froth was forced from his mouth, and the blood ceased to flow from the temporal artery. The froth worked up after his breathing stopped, and he died, as I was attempting to raise his head, without a groan or a convulsion. —*Cheyne on Apoplexy*, p. 92." 87.

This case is very interesting, and deserves to be attentively and critically examined. It possesses few of the characters of genuine Apoplexy. The patient was not unconscious or insensible, until shortly before death. He complained, it will be observed, of a pain under the left nipple, and uneasiness in the left shoulder; he became faint; his lips were livid, and his extremities cold. The symptoms seem to have become decidedly worse after the bleeding. As we have intimated above, we strongly suspect that this was a case of *cardiac* rather than of *cerebral* disease.

To the distinguished author of the Dictionary of Practical Medicine—would that the ten years ago promise of a speedy completion of this great work were redeemed!—we owe the following case, in which the recovery from a well marked-apoplectic seizure took place without blood-letting.

CASE.—*Apoplectic seizure in a stage-coach; profound stupor and immobility; pulse slow and natural; cold affusion on the head, and stimulants to the nostrils; returning consciousness; use of purgatives and subsequently of bleeding; recovery.*

"Travelling in the summer, in one of the short stages, I sat opposite an aged and corpulent man, who suddenly lost his consciousness and power of motion. His countenance became first pale, then bloated and inexpressive, his breathing slow and slightly stertorous; all his muscles completely relaxed, and he fell, in a few seconds, upon those sitting around him. We were only a few doors from a chemist's shop; the coach was stopped, and he was carried thither. He was now profoundly apoplectic; a copious perspiration flowed from his face and forehead, the veins of which were distended, and all his senses were completely abolished. There was no sign of hemiplegia, but there was general and complete loss of motion and sensation. His neckcloth having been removed, the pulsation of the carotids was found to be slow, and of natural strength and fulness.

Whilst he was held in a sitting posture, in a chair, cold water was poured gently over his head from a sponge, and his head frequently sponged with it; volatile salts also were held for a short time, and at intervals to his nostrils. The power of deglutition was at this time abolished, so that it was impossible to administer a draught, consisting of sp. ammon. arom. and camphor mixture, which was prescribed. In a very few minutes his consciousness returned, he took the draught, and, in a short time afterwards, he walked to a coach, in which I accompanied him home. He now complained only of very slight confusion of ideas, with scarcely any headache, but his carotids beat more firmly. One full blood-letting and an active purgative, were now directed. The next day he was perfectly well, and has continued so." 134.

Portal relates that Turgot had a frightful attack of apoplexy, at the moment of his experiencing pains in the feet announcing the approach of gout. He was of a strong constitution, very fat, with a red countenance, and was then about 50 years of age. He was bled in the foot; this diminished the severity of the apoplexy; sinapisms, a second bleeding in the foot, and some aperient medicine removed it.

Lieutaud relates the following case, which did well under similar mild treatment.

CASE.—*Apoplectic attack after a meal; the mind had been much disquieted; returning consciousness; paralysis of the arm and face; leeches applied to the anus, blisters to the legs and arm, and purgatives administered; recovery.*

"M. l'Abbé de Boismont, about 66 years of age, extremely sensitive and passionate, for a long time complained of cramp in the muscles of the legs, especially of a night, and several times had felt a tingling in the fingers and toes. One day, after a meal, having been troubled with some mental affection, he was attacked with true apoplexy. I was summoned, but did not arrive until after the patient had recovered his recollection; he still had complete paralysis of the left upper extremity, and the mouth was drawn to the right side. Pulse full, countenance flushed. As he had formerly suffered from piles, I ordered leeches to the anus. This bleeding was repeated, and blisters were applied to the legs and one of the arms, Laxative draughts were administered. Afterwards the water of Balaruc, and other tonics. The patient improved, and from the great attention that was paid him, lived some time; he died with marasmus." 153.

The following two cases are adduced by Dr. Hull, in his work on blood to the head, to shew that apoplectiform symptoms may be induced, in certain states of the body, by excessive purgation.

"S——, foreman of a mill; stout; ventri deditus: was under the care of a physician for disorder in the liver, which was treated with mercury. On the 8th of February, 1841, this had produced a hypercatharsis so prodigious, that his family surgeon found him nearly bereft of all power and all *pulse*. By judicious attention he rallied partially; and twelve hours after was visited by myself in consultation. I found him presenting all the cerebral symptoms of imminent apoplexy: lethargic, flushed. Yet the great debility and velocity of his pulse forbad an appeal to any other system than the nervous. Accordingly, mustard cataplasms were applied to his feet. But he went on into all the degrees of apoplexy, and died within twelve hours more."

"C——, a stout, unwieldy lady, near 70, fell into a coma whilst walking out after dinner. Her surgeon administered an emetic, let blood twice and freely, and ordered a purgative. Mustard cataplasms. In a few hours consciousness was displayed: in a few days her intellect was restored. But her power of walking was not regained for months. I was called to this patient within two years, and found her apoplectic and moribund. In her usual health in the morning, she had taken a purgative, which had produced a hypercatharsis. After the evacuations she is struck with apoplexy. Emptied vessels, great debility: yet blood determined fatally to the head!" 165.

However injurious hypercatharsis may occasionally be, we should ever remember that no remedies are on the whole more useful in preventing a threatened invasion of apoplexy, or in recovering patients from the less formidable attacks of the disease, than active purgatives. The immediate use of strong sinapisms to the neck and of stimulating enemata should, perhaps, never be omitted in any case. We have already said that diligent enquiry should invariably be made whether the patient had been eating freely before the attack; for, whenever there is reason to believe that the stomach is loaded with food, an emetic should forthwith be administered. One or two of the cases, which have been related, afford striking proof of the efficacy of such treatment.

In taking leave of Mr. Copeman's work, we doubt not that our readers will agree with us, that there is a good deal of judicious and really useful instruction in its pages. We trust that he will be encouraged to prosecute his researches on the subject which he has chosen, and ere long give the public the benefit of his matured investigation.

